# Planetary Health Diet and the Gut–Liver–Brain Axis: Implications for MASLD and Cognitive Aging

**DOI:** 10.3390/nu18193275

**Published:** 2026-10-05

**Authors:** Hairong Cai, Chulin Chen, Huiling Cai, Deju Zhang, Qiuping Huang

**Affiliations:** 1 The Second Clinical College, Guangzhou University of Chinese Medicine, Guangzhou 510120, China; caihairong@gzucm.edu.cn (H.C.); 18719387847@163.com (C.C.); a1678638738@163.com (H.C.); 2 School of Nursing, Guangzhou University of Chinese Medicine, Guangzhou 510006, China; 3 Food and Nutritional Sciences, School of Biological Sciences, The University of Hong Kong, Pokfulam Road, Hong Kong; u3005757@connect.hku.hk

**Keywords:** Planetary Health Diet, gut–liver–brain axis, MASLD, cognitive aging, dementia, gut microbiome, short-chain fatty acids, bile acids, intestinal barrier, EAT–Lancet

## Abstract

**Background**: Metabolic dysfunction-associated steatotic liver disease (MASLD) and cognitive aging are prevalent, interrelated conditions that share gut-derived pathomechanisms. The Planetary Health Diet (PHD), a sustainable plant-forward framework proposed by the EAT–Lancet Commission, may offer dual-target benefits for hepatic and neurological health through the gut–liver–brain axis. **Methods**: A structured narrative literature search of PubMed, Web of Science, and Scopus (2015–2026) was conducted to identify observational, mechanistic, and interventional studies addressing PHD adherence in relation to MASLD, cognitive aging, and gut–liver–brain axis pathways. **Results**: Epidemiological evidence from large cohorts links higher PHD adherence to lower MASLD prevalence and severity, slower cognitive decline, reduced dementia incidence, and more favorable Alzheimer’s disease neuropathology. Mechanistically, PHD components may modulate gut microbiota composition and diversity, enhance short-chain fatty acid production, regulate bile acid signaling through farnesoid X receptor (FXR) and Takeda G-protein-coupled receptor 5 (TGR5), reinforce intestinal barrier integrity, and attenuate metabolic endotoxemia and neuroinflammation. **Conclusions**: Although current evidence remains predominantly observational, the PHD is a promising dietary framework for simultaneously supporting liver and brain health. Future research should prioritize randomized controlled trials integrating liver imaging, cognitive assessment, microbiome profiling, and culturally adapted dietary interventions.

## 1. Introduction

Metabolic dysfunction-associated steatotic liver disease (MASLD) and age-related cognitive decline constitute two of the most formidable global health challenges in the twenty-first century, imposing enormous burdens on healthcare systems, caregivers, and societies worldwide. Using the historical NAFLD definition, a 2022 systematic review and meta-analysis estimated a pooled global prevalence of 32.4% (95% CI, 29.9–34.9), with prevalence reaching 37.8% (95% CI, 32.4–43.3) among studies conducted in 2016 or later [1]. These estimates provide temporal context for the current global burden of MASLD but should not be interpreted as a direct prevalence estimate under the 2023 MASLD diagnostic nomenclature. The condition encompasses a histological spectrum ranging from simple steatosis through metabolic dysfunction-associated steatohepatitis (MASH), progressive fibrosis, cirrhosis, and hepatocellular carcinoma, and is closely intertwined with obesity, insulin resistance, type 2 diabetes (T2D), dyslipidemia, and cardiovascular disease [2,3]. Concurrently, the global burden of dementia continues to escalate, with the Global Burden of Disease Study estimating that over 55 million people were living with dementia in 2019, a figure projected to surpass 150 million by 2050 [4,5]. The 2024 Lancet Commission on dementia prevention identified 14 modifiable risk factors, several of which—including diabetes, obesity, physical inactivity, hypertension, and depression—overlap substantially with MASLD risk factors [5].

The convergence of these two epidemics is not coincidental. MASLD and cognitive aging share a constellation of biological pathways, including insulin resistance, chronic low-grade systemic inflammation, oxidative stress, vascular dysfunction, dysregulated lipid metabolism, altered bile acid signaling, and gut microbiota dysbiosis [6,7,8]. The gut–liver axis, sustained by portal circulation, bile acid enterohepatic cycling, microbial metabolites, and immune signaling, is now recognized as a central mediator of hepatic inflammation and metabolic disease progression [9]. The gut–brain axis encompasses neural, endocrine, immune, and metabolic pathways through which gut-derived signals modulate neuroinflammation, blood–brain barrier integrity, stress responses, and cognition [10]. These overlapping mechanistic frameworks provide a compelling rationale for investigating dietary patterns that may simultaneously influence hepatic and neurological outcomes through what can be conceptualized as a gut–liver–brain axis.

Against this backdrop, the Planetary Health Diet (PHD), first proposed by the EAT–Lancet Commission in 2019, has emerged as a landmark dietary framework that integrates human health optimization with environmental sustainability [11]. The PHD is characterized by high intake of whole grains, legumes, nuts, vegetables, and fruits; moderate consumption of fish, poultry, and dairy; and limited intake of red meat, processed foods, and added sugars [12,13]. Adherence to PHD-like dietary patterns has been associated with reduced total mortality [14,15,16], lower cardiovascular disease risk [17,18], decreased type 2 diabetes incidence [19,20], and decreased incidence of depression and anxiety [21] across diverse populations. More recently, a rapidly expanding body of evidence has begun to link PHD adherence with both MASLD-related outcomes [22,23,24] and cognitive aging trajectories [25,26,27,28].

Despite these advances, no review to date has systematically integrated the evidence on PHD in relation to both MASLD and cognitive aging through the lens of the gut–liver–brain axis. This narrative review aims to: (1) define the PHD and summarize evidence linking PHD adherence to MASLD risk and severity; (2) review the evidence relating PHD to cognitive decline, dementia risk, and brain pathology; (3) elucidate the mechanistic pathways through the gut–liver–brain axis by which PHD components may influence both hepatic and neurological health; and (4) discuss clinical implications, limitations, and priorities for future research.

Importantly, the current evidence base should be interpreted in light of its methodological limitations. Most studies examining PHD adherence in relation to MASLD or cognitive outcomes are observational, and direct PHD-specific intervention studies remain scarce. Moreover, evidence supporting several proposed gut–liver–brain mechanisms is derived from studies of individual dietary components, other plant-forward dietary patterns, or preclinical experimental models rather than from interventions testing the complete PHD. Accordingly, the associations summarized in this review should not be interpreted as establishing causality, and mechanistic plausibility is distinguished throughout from directly demonstrated clinical effects.

## 2. Methods

This narrative review used a structured, non-systematic literature synthesis approach to examine evidence connecting the Planetary Health Diet with MASLD, cognitive aging, and the gut–liver–brain axis. A structured literature search was conducted across three electronic databases: PubMed/MEDLINE, Web of Science Core Collection, and Scopus. The search strategy employed five conceptual modules combined with Boolean operators: (1) “Planetary Health Diet” OR “EAT–Lancet diet” OR “EAT–Lancet reference diet” OR “Planetary Health Diet Index”; (2) “MASLD” OR “NAFLD” OR “metabolic dysfunction-associated steatotic liver disease” OR “fatty liver”; (3) “cognitive aging” OR “cognitive decline” OR “dementia” OR “Alzheimer” OR “cognitive impairment” OR “brain health”; (4) “gut microbiome” OR “gut microbiota” OR “gut–liver axis” OR “gut–brain axis” OR “gut–liver–brain axis” OR “intestinal barrier” OR “short-chain fatty acids” OR “bile acids” OR “endotoxemia”; (5) “dietary pattern” OR “diet quality” OR “plant-based diet” OR “Mediterranean diet” OR “MIND diet”. Studies were eligible for inclusion if they were published in English between January 2015 and April 2026, involved human participants or provided mechanistic insights from well-designed preclinical studies with clear human relevance, and addressed at least one of the review’s core topics: PHD adherence measurement, PHD associations with MASLD or liver outcomes, PHD associations with cognitive or dementia outcomes, or mechanistic pathways linking dietary patterns to liver and brain health through the gut–liver–brain axis.

Studies were excluded if they focused exclusively on pediatric populations, were limited to animal models without a clear connection to human physiology or clinical relevance, or were available only as conference abstracts without peer-reviewed full-text publication. Hairong Cai, Chulin Chen, and Huiling Cai participated in literature identification and study eligibility assessment. Potentially relevant records were evaluated against the predefined inclusion and exclusion criteria, with particular attention to study population, dietary exposure, clinical or mechanistic outcomes, and relevance to the PHD–MASLD–cognitive aging framework. Any uncertainty regarding study eligibility was resolved through discussion among the authors. Reference lists of relevant reviews, systematic reviews, and meta-analyses were additionally hand-searched to identify potentially eligible studies not captured by the electronic search.

Because this work was designed as a structured narrative review rather than a systematic review, no formal risk-of-bias or methodological quality assessment using standardized instruments was undertaken. Instead, evidence was interpreted according to study design, population, sample size, exposure and outcome assessment, degree of covariate adjustment, consistency across studies, and whether the evidence originated from human observational studies, intervention studies, animal models, or in vitro experiments. These distinctions were considered throughout the synthesis to avoid assigning equivalent evidentiary weight to epidemiological associations and mechanistic findings. Accordingly, the synthesis emphasizes the hierarchy and context of evidence rather than treating observational associations, intervention findings, and preclinical mechanistic evidence as equivalent forms of support.

## 3. The Planetary Health Diet: Concept, Reference Targets, and Adherence Indices

The EAT–Lancet Commission introduced the EAT–Lancet reference diet in 2019 as a quantitative dietary framework intended to support human health while remaining compatible with global environmental boundaries. The reference diet specifies target intake ranges for major food groups, emphasizing vegetables, fruits, whole grains, legumes, nuts, and unsaturated plant oils; allowing moderate amounts of fish, poultry, eggs, and dairy; and recommending substantially lower consumption of red meat, added sugars, and saturated fats [11,12]. Importantly, the EAT–Lancet reference diet should be distinguished from the broader term “Planetary Health Diet” (PHD). In the contemporary literature, PHD is commonly used to describe a plant-forward dietary pattern inspired by the principles and food-group targets of the EAT–Lancet framework, but its implementation may vary across populations, cultural settings, and epidemiological studies. Thus, the PHD represents the broader dietary concept, whereas the EAT–Lancet reference diet provides the original quantitative benchmark from which many PHD operationalizations have been derived.

Because the EAT–Lancet reference diet provides food-group targets rather than a single universally prescribed epidemiological score, several adherence indices have been developed to operationalize the PHD in observational research. Among these, Cacau et al. developed and validated the Planetary Health Diet Index (PHDI), which comprises 16 dietary components and yields a total score ranging from 0 to 150 points, with higher scores indicating closer adherence to the dietary principles of the EAT–Lancet framework [11]. The PHDI incorporates adequacy, optimum, ratio, and moderation components to capture both greater consumption of recommended plant-based foods and restricted consumption of foods for which lower intake is encouraged. Importantly, the PHDI represents one operational measure of adherence rather than the definition of the PHD itself. Other investigators have operationalized adherence to the EAT–Lancet framework using alternative scoring systems, including indices developed by Stubbendorff et al., Knuppel et al., and Kesse-Guyot et al. These approaches differ in the number and definition of dietary components, whether intake is assessed against absolute gram-based targets or population-specific distributions, the treatment of intermediate intake levels, and the weighting assigned to individual food groups. Consequently, individuals classified as having high PHD adherence according to one index may not be classified identically under another. Throughout this review, we therefore use “PHD adherence” as an umbrella term when discussing the broader literature and specify the particular adherence index whenever this information is available [29]. This distinction is especially important when comparing effect estimates across epidemiological studies.

The original reference diet specifies approximate target intakes for major food groups—for example, approximately 300 g/day of vegetables, 200 g/day of fruit, 232 g/day of whole grains, 75 g/day of legumes, 50 g/day of nuts, and substantially lower quantities of red meat and added sugars. These quantities serve as reference targets rather than constituting a universal PHD adherence score.

The PHD also shares substantial features with other evidence-based healthy dietary patterns while retaining a distinct conceptual foundation. Like the Mediterranean diet, it emphasizes whole grains, vegetables, fruits, legumes, nuts, unsaturated fats, and relatively limited red and processed meat. However, the original EAT–Lancet reference diet provides explicit quantitative target ranges for major food groups and incorporates environmental sustainability into its underlying design. Compared with the DASH diet, the PHD places relatively greater emphasis on plant protein sources, legumes, nuts, and environmental considerations, whereas DASH was primarily developed for blood pressure reduction and emphasizes sodium restriction and low-fat dairy. As shown in Table 1, the MIND diet, in contrast, was specifically designed around foods hypothesized to support cognitive health, including berries and green leafy vegetables [12,13]. These differences in original purpose and scoring should be considered when comparing their associations with MASLD or cognitive outcomes. An important consideration, particularly in older adults, is that population-level adherence to a strongly plant-forward dietary framework does not automatically guarantee nutritional adequacy at the individual level. Modeling studies have raised concerns regarding potentially inadequate intakes of vitamin B12, calcium, iron, and zinc under strict implementation of the EAT–Lancet reference diet, particularly where fortified foods or supplementation are limited [13]. Protein quantity and quality also warrant attention in older populations because inadequate protein and energy intake may contribute to loss of muscle mass, functional decline, and sarcopenia. Accordingly, implementation of PHD principles in older adults should emphasize adequate total protein intake and complementary protein sources, including legumes, soy products, nuts, seeds, whole grains, dairy, eggs, fish, or other culturally appropriate foods where applicable. Individualized assessment of vitamin B12, iron, calcium, zinc, and vitamin D status, followed by fortification or supplementation when clinically indicated, may be necessary. These considerations are discussed further in the clinical implications section.

**Figure 1:**
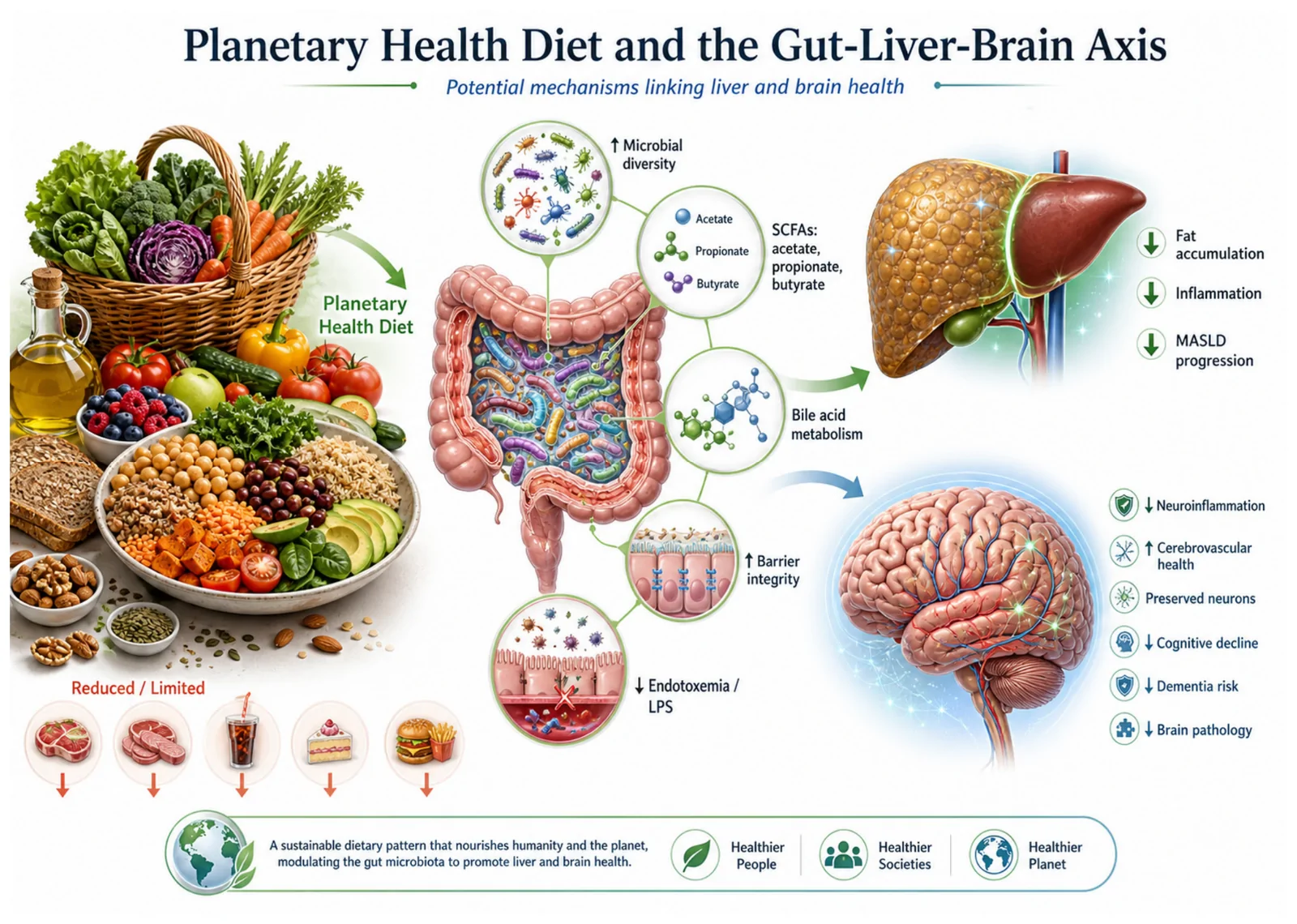
Overall conceptual framework of the Planetary Health Diet, MASLD, cognitive aging, and the gut–liver–brain axis. PHD, Planetary Health Diet; MASLD, metabolic dysfunction-associated steatotic liver disease; SCFAs, short-chain fatty acids; FXR, farnesoid X receptor; TGR5, Takeda G-protein-coupled receptor 5; LPS, lipopolysaccharide.

Figure 1 provides an integrated conceptual overview of how the PHD may intersect with MASLD and cognitive aging through shared gut–liver–brain pathways. The individual epidemiological and mechanistic evidence streams underlying this framework are examined in detail in the subsequent sections.

## 4. MASLD: Definition, Pathophysiology, and Global Burden

The nomenclature of fatty liver disease underwent a paradigm shift in 2023 when a multisociety Delphi consensus process replaced the term non-alcoholic fatty liver disease (NAFLD) with metabolic dysfunction-associated steatotic liver disease (MASLD) [32]. This renaming reflected a fundamental reconceptualization: whereas NAFLD was defined by the exclusion of other liver diseases (particularly alcohol), MASLD is defined by the inclusion of at least one cardiometabolic risk factor in the presence of hepatic steatosis, thereby acknowledging the central role of metabolic dysfunction in disease pathogenesis [2,32]. The MASLD definition encompasses a disease spectrum that progresses from simple steatosis (now termed MASLD) through metabolic dysfunction-associated steatohepatitis (MASH), progressive fibrosis, compensated and decompensated cirrhosis, and ultimately hepatocellular carcinoma [3,33].

The global prevalence of MASLD has risen in parallel with the obesity and T2D pandemics, now estimated at approximately 30% of the adult population worldwide, with regional variations reflecting differences in metabolic risk factor prevalence, genetic susceptibility, dietary patterns, and physical activity levels [1]. In the United States, MASLD prevalence exceeds 35% among adults, while rates in East Asian and South Asian populations have risen sharply over the past two decades [34]. The condition is particularly prevalent among individuals with T2D, where prevalence estimates range from 55% to 75%, and among those with obesity, where rates may exceed 80% [35]. The economic burden is substantial, with MASLD-associated healthcare costs projected to increase dramatically as the affected population ages and disease progresses to more advanced stages.

The pathophysiology of MASLD is complex and multifactorial, involving the interplay of metabolic, genetic, environmental, and gut-derived factors. The traditional “two-hit” model has been superseded by a more nuanced “multiple parallel hits” framework that recognizes the simultaneous contributions of lipotoxicity, insulin resistance, mitochondrial dysfunction, endoplasmic reticulum stress, inflammatory cascades, and gut-derived signals to hepatocyte injury and disease progression [36,37]. Lipotoxicity, driven by excessive free fatty acid accumulation and toxic lipid species, activates inflammatory pathways including nuclear factor kappa B (NF-κB) and c-Jun *N*-terminal kinase signaling, promoting hepatocyte apoptosis and stellate cell activation [38]. Insulin resistance, a central feature of metabolic syndrome, promotes hepatic de novo lipogenesis while simultaneously impairing fatty acid β-oxidation, creating a net positive lipid balance in the liver [36].

Extrahepatic manifestations of MASLD have emerged as a critical dimension of disease burden. Cognitive impairment and dementia have increasingly been reported among the extrahepatic conditions associated with MASLD. However, this relationship should be interpreted cautiously because MASLD and cognitive aging share a substantial cardiometabolic and vascular risk architecture. Obesity, insulin resistance, type 2 diabetes, hypertension, dyslipidemia, physical inactivity, and systemic vascular disease are common determinants of both hepatic steatosis and cognitive decline and may therefore confound, mediate, or partly account for the observed association. Accordingly, epidemiological associations between MASLD and cognitive outcomes do not by themselves establish a direct liver-to-brain causal pathway. Rather, current evidence supports a model in which shared metabolic and vascular susceptibility may coexist with additional liver-derived, inflammatory, and gut-mediated signals that potentially contribute to brain aging [7,8,35].

Distinguishing confounding from biological mediation is particularly important in this context. For examplein Table 2, diabetes, obesity, hypertension, and atherosclerotic disease may precede and independently contribute to both MASLD and cognitive impairment, whereas systemic inflammation, altered bile acid signaling, gut-derived metabolites, and hepatic metabolic dysfunction may represent potential mediating pathways after MASLD has developed. Existing observational studies generally cannot fully disentangle these pathways. Therefore, the mechanistic relationships summarized below should be regarded as biologically plausible and, in several instances, hypothesis-generating rather than as established causal mechanisms in humans.

Taken together, the current literature supports an epidemiological association between MASLD and adverse cognitive outcomes, but the extent to which this association reflects shared cardiometabolic and vascular risk, versus liver-specific biological effects, remains unresolved. This distinction is central to interpreting the potential role of dietary patterns such as the PHD in simultaneously influencing liver and brain health.

## 5. Evidence Linking the Planetary Health Diet to MASLD

The mechanistic rationale for investigating PHD in the context of MASLD is compelling. The PHD’s characteristic features—high fiber content, abundant polyphenols from diverse plant sources, low saturated fat, minimal processed food, and limited added sugars—directly target several of the core pathogenic processes underlying MASLD, including hepatic de novo lipogenesis, insulin resistance, systemic inflammation, and gut dysbiosis. This section reviews the rapidly expanding observational evidence linking PHD adherence to MASLD risk and severity.

Like Table 3, the first large-scale metabolomic investigation of this relationship was conducted by Wu et al. using data from the UK Biobank [22]. In a prospective analysis of over 100,000 participants followed for a median of approximately 12 years, higher adherence to the EAT–Lancet reference diet, assessed using the EAT–Lancet diet index, was associated with a 21% lower risk of incident MASLD (hazard ratio [HR] 0.79, 95% confidence interval [CI] 0.72–0.87) comparing the highest with the lowest adherence quartile. Critically, this study identified a metabolomic signature comprising 81 metabolites that partially mediated the diet-MASLD association, providing mechanistic insight into the biological pathways involved. Furthermore, the inverse association was not significantly modified by MASLD polygenic risk score, suggesting that the observed relationship was present across different levels of genetic susceptibility [22].

A landmark multicohort analysis by Zhang et al. extended these findings across four independent cohorts from different geographic regions [23]. The study demonstrated that higher adherence to the EAT–Lancet reference diet was consistently associated with lower MASLD prevalence and reduced disease severity, as assessed by non-invasive markers including the fatty liver index and liver enzyme elevations. The observation of broadly similar associations across populations with different dietary cultures supports the external consistency of the findings. However, cross-cohort replication does not eliminate residual confounding, particularly because PHD adherence tends to cluster with other favorable metabolic, behavioral, and socioeconomic characteristics.

Cross-sectional analyses from nationally representative datasets have corroborated these prospective findings. Qiu et al. analyzed data from the National Health and Nutrition Examination Survey (NHANES) 2005–2018 and reported that higher PHDI scores were associated with significantly lower odds of MASLD after adjustment for demographic, lifestyle, and metabolic confounders [24]. Similarly, Doustmohammadian et al. confirmed the inverse PHDI-MASLD association in a separate NHANES analysis, adding to the consistency of the cross-sectional evidence base [42]. A GBD-based analysis by Liu et al. integrated Global Burden of Disease data with the Global Dietary Database and NHANES to examine the PHDI-MASLD relationship at the population level, finding that countries with higher estimated PHD adherence had lower MASLD prevalence [43].

The co-occurrence of MASLD with T2D represents a particularly high-risk phenotype. Zhang et al. examined the relationship between EAT–Lancet diet adherence, T2D, MASLD, and their co-occurrence using data from both the UK Biobank and a Chinese biopsy-proven MASLD cohort [44]. Higher diet adherence was independently associated with lower risks of T2D, MASLD, and their co-occurrence, suggesting that the PHD may address the shared metabolic substrate underlying both conditions. In a broader investigation of liver outcomes, Wu et al. reported that EAT–Lancet diet adherence was associated with reduced risk of multiple chronic liver diseases in a prospective UK Biobank analysis, with associations remaining significant after genetic risk stratification [45].

These observational findings nevertheless require cautious interpretation because residual confounding remains an important limitation. Higher adherence to the PHD is frequently accompanied by lower adiposity, greater physical activity, lower smoking prevalence, better cardiometabolic profiles, higher educational attainment, greater health literacy, and higher socioeconomic position. Several of these characteristics are themselves strongly associated with MASLD risk. Although most cohort analyses adjust for major demographic, metabolic, and lifestyle covariates, adjustment may be incomplete because adiposity, diabetes severity, physical activity, smoking, alcohol use, healthcare access, and socioeconomic conditions are measured with varying precision and may change over time. Moreover, some variables, particularly body weight, insulin resistance, and diabetes, may function simultaneously as confounders and intermediate factors on the pathway between diet and MASLD, making statistical adjustment conceptually complex. Therefore, the inverse associations observed across PHD studies should not be interpreted as estimates of a purely independent causal effect of the dietary pattern.

A related concern is the healthy-user effect: individuals who adhere closely to a PHD-like pattern may also engage in multiple health-promoting behaviors that are difficult to capture fully in epidemiological models. Consequently, even well-adjusted prospective associations may retain some degree of behavioral and socioeconomic confounding.

The role of broader dietary patterns in MASLD progression was examined by Li et al., who conducted a systematic review of plant-forward dietary patterns and liver disease outcomes [46]. Their synthesis indicated that plant-forward patterns, including Mediterranean and PHD-like patterns, were associated with slower progression from steatosis to advanced liver disease, supporting the hypothesis that dietary quality is a modifiable determinant of MASLD natural history. Several mechanisms could plausibly contribute to the observed PHD–MASLD association, including improved insulin sensitivity, reduced inflammatory signaling, favorable modulation of gut microbial metabolism, altered hepatic de novo lipogenesis, and improved lipid profiles. However, most of these pathways are inferred from studies of individual dietary components, related dietary patterns, or experimental models, and have not yet been demonstrated as mediators in controlled PHD-specific intervention studies.

**Figure 2:**
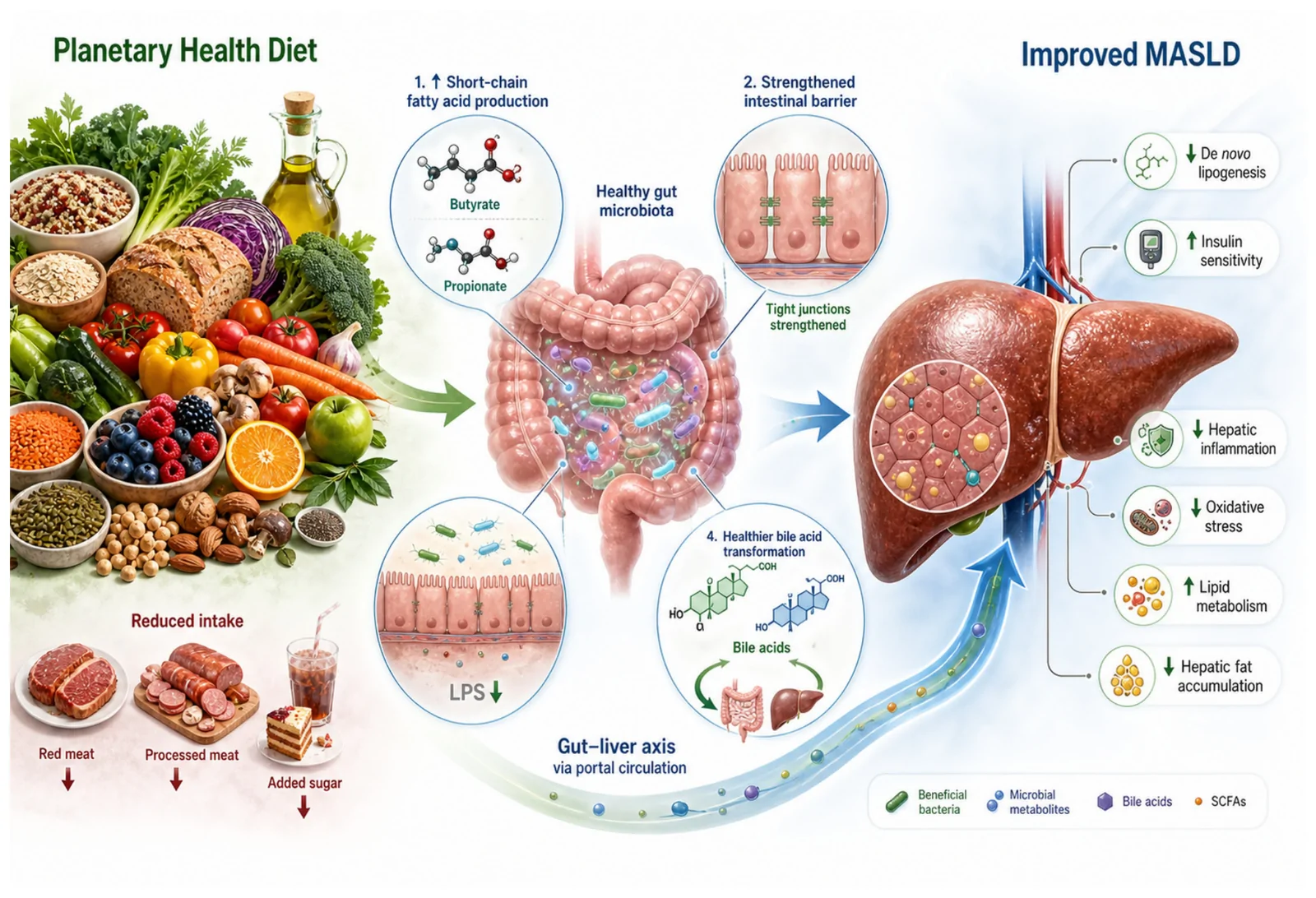
Mechanisms by which the Planetary Health Diet may improve MASLD through the gut–liver axis. PHD, Planetary Health Diet; MASLD, metabolic dysfunction-associated steatotic liver disease; SCFAs, short-chain fatty acids; FXR, farnesoid X receptor; TGR5, Takeda G-protein-coupled receptor 5; LPS, lipopolysaccharide; MASH, metabolic dysfunction-associated steatohepatitis.

Figure 2 illustrates the key mechanisms by which the PHD may improve MASLD outcomes through the gut–liver axis, including modulation of gut microbiota composition, enhancement of SCFA production, bile acid signaling through FXR and TGR5, reinforcement of intestinal barrier integrity, and attenuation of metabolic endotoxemia.

**Table 3:** Summary of studies examining the association between Planetary Health Diet adherence and MASLD outcomes.

Study	Population	Design	Key Finding	Outcome Measure
Wu et al. [22]	UK Biobank, *n* > 100,000	Prospective cohort, ~12 y follow-up	21% lower MASLD risk (HR 0.79) in highest vs. lowest EAT–Lancet quartile; 81 metabolites identified	Incident MASLD, metabolomic signature
Zhang et al. [23]	4 international cohorts	Multicohort analysis	Consistent inverse association across populations with diverse dietary cultures	MASLD prevalence, liver enzyme elevations
Qiu et al. [24]	NHANES 2005–2018	Cross-sectional	Higher PHDI associated with lower MASLD odds	MASLD by fatty liver index
Zhang et al. [44]	UK Biobank + Chinese MASLD cohort	Prospective + cross-sectional	Protective against T2D, MASLD, and co-occurrence	T2D, MASLD, co-occurrence
Wu et al. [45]	UK Biobank	Prospective cohort	Reduced risk of multiple chronic liver diseases	Multiple liver disease endpoints
Liu et al. [43]	GBD + Global Dietary Database + NHANES	Ecological + cross-sectional	Countries with higher PHD adherence show lower MASLD prevalence	Population-level MASLD prevalence
Doustmohammadian et al. [42]	NHANES	Cross-sectional	Inverse PHDI-MASLD association confirmed	MASLD by imaging/biochemical criteria
Li et al. [46]	Multiple populations	Systematic review	Plant-forward patterns associated with slower liver disease progression	Liver disease histological progression

## 6. Cognitive Aging: Mechanisms and Dietary Modifiable Risk Factors

Cognitive aging encompasses a continuum from normal age-related changes in processing speed, episodic memory, and executive function through mild cognitive impairment (MCI) to clinical dementia, including Alzheimer’s disease (AD), vascular dementia, and mixed-pathology presentations [4,47]. This continuum is shaped by a complex interplay of genetic susceptibility (most notably APOE ε4 carrier status), biological aging processes, vascular and metabolic risk factors, lifestyle behaviors, and environmental exposures. The biological processes underlying cognitive aging include neuronal autophagy, mitochondrial dysfunction, cellular senescence, epigenetic alterations, cerebrovascular dysfunction, neuroinflammation, and accumulation of pathological proteins including amyloid-β and hyperphosphorylated tau [47].

The identification of modifiable risk factors for dementia has been a transformative development in the field. The landmark 2024 Lancet Commission on dementia prevention, intervention, and care estimated that approximately 45% of dementia cases worldwide are attributable to 14 potentially modifiable risk factors: low education, hearing loss, hypertension, excessive alcohol consumption, obesity, traumatic brain injury, smoking, depression, social isolation, physical inactivity, air pollution, diabetes, untreated vision loss, and high low-density lipoprotein cholesterol [5]. This attributable fraction provides a powerful impetus for preventive strategies targeting these factors, among which diet quality has attracted substantial attention as a potentially modifiable determinant of late-life cognitive trajectories [47,48].

The Mediterranean diet has the most extensive evidence base among dietary patterns studied in relation to cognitive outcomes. As shwon in Table 4, meta-analyses of prospective cohort studies suggest that higher Mediterranean diet adherence is associated with a 20–30% reduction in dementia risk [49,50]. The proposed mechanisms include anti-inflammatory effects of polyphenols and omega-3 fatty acids, improved vascular function, reduced insulin resistance, favorable gut microbiome modulation, and reduced oxidative stress. The MIND diet, a hybrid of Mediterranean and DASH dietary principles specifically targeting foods hypothesized to be neuroprotective, was developed by Morris et al. and initially associated with a 53% reduction in AD risk in the Rush Memory and Aging Project [30]. However, the landmark MIND randomized controlled trial (RCT), enrolling 604 participants over 3 years, found that both the MIND diet intervention and the control diet (which received mild caloric restriction counseling) groups experienced cognitive improvement, with a non-significant between-group difference [31]. This result highlighted the difficulty of demonstrating diet-specific cognitive benefits beyond general healthy-eating advice in an RCT setting.

Omega-3 fatty acids, particularly docosahexaenoic acid (DHA) and eicosapentaenoic acid (EPA), represent one of the most extensively studied nutritional components in relation to brain health. DHA is a major structural component of neuronal membranes and gives rise to neuroprotectin D1 and other specialized pro-resolving mediators with anti-inflammatory and neuroprotective properties [51]. Observational studies consistently associate higher omega-3 intake or blood levels with slower cognitive decline, though RCT evidence remains inconsistent. The gut microbiome has emerged as a potential mediator of dietary effects on cognition, with gut-derived metabolites including short-chain fatty acids, tryptophan metabolites, and bile acids modulating neuroinflammation and blood–brain barrier function [10,48].

**Table 4:** Dietary patterns and cognitive outcomes: comparison of evidence.

Pattern	Key Components	Cognitive Evidence	Mechanisms
Mediterranean	Olive oil, vegetables, fruits, whole grains, legumes, fish, moderate wine	Meta-analyses: 20–30% dementia risk reduction [49,50]; slower cognitive decline	Anti-inflammatory polyphenols, omega-3, improved vascular function, gut microbiome
MIND	Mediterranean + DASH hybrid; green leafy vegetables, berries, nuts, olive oil, whole grains, fish	Initial cohort: 53% AD risk reduction [30]; MIND RCT: non-significant between-group effect [31]	Targets neurodegeneration-specific foods; polyphenols, carotenoids, omega-3
DASH	Low sodium, fruits, vegetables, whole grains, low-fat dairy, limited saturated fat	Some evidence for slower cognitive decline; less studied than Mediterranean	Blood pressure reduction, vascular protection
Plant-based	Vegetables, fruits, legumes, whole grains, nuts; limited/no animal products	Variable evidence; quality of plant-based diet matters [52]	Fiber, polyphenols, microbiome modulation; risk of B12/iron inadequacy
Planetary Health Diet	Whole grains, legumes, nuts, vegetables, fruits; limited red/processed meat [11]	Growing observational evidence (see Table 5)	Combined anti-inflammatory, vascular, metabolic, and gut-mediated pathways

## 7. Evidence Linking the Planetary Health Diet to Cognitive Aging

The emerging literature on the PHD and cognitive aging encompasses several distinct outcome domains, including cognitive performance, longitudinal cognitive decline, subjective cognitive complaints, incident dementia and Alzheimer’s disease (AD), and structural or neuropathological brain markers. These outcomes should not be treated as interchangeable because they represent different stages of the cognitive aging continuum and are supported by different levels of evidence. At present, the PHD-specific evidence base is dominated by observational studies; therefore, the findings summarized below are interpreted as associations rather than proof of a preventive or therapeutic effect. Biological mechanisms involving inflammation, vascular function, insulin sensitivity, and gut-derived metabolites provide plausibility for these associations but do not establish causality.

### 7.1. Cognitive Performance and Longitudinal Cognitive Decline

Several prospective studies have examined PHD adherence in relation to cognitive performance and longitudinal cognitive trajectories rather than clinically diagnosed dementia. In the ELSA–Brazil cohort, Gomes Gonçalves et al. followed 11,737 participants for approximately 8 years and reported that higher PHD adherence was associated with slower decline in memory and global cognitive performance [25,26]. Evidence of a graded association was observed across adherence levels, although the magnitude of the association differed according to income, indicating potential socioeconomic effect modification. In a cohort of Chinese older adults, Tang et al. similarly reported that greater adherence to the PHD was prospectively associated with a slower rate of cognitive decline across repeated assessments [27].

Related evidence has emerged from other population-based cohorts. In three large US prospective cohorts comprising 159,347 participants, Chen et al. compared several healthy dietary patterns and found that higher PHDI scores were associated with a lower likelihood of subjective cognitive decline [53]. Because subjective cognitive decline differs from objectively measured cognitive deterioration and clinical dementia, this finding should be interpreted as evidence relating to perceived cognitive change rather than a dementia endpoint. Zhang et al. further reported that mid-life PHD adherence was associated with better late-life cognitive performance among participants in the Singapore Chinese Health Study, with stronger associations observed among APOE ε4 non-carriers [54]. Cross-sectional analyses from the UK Biobank have likewise linked greater EAT–Lancet diet adherence with more favorable cognitive aging measures [55]. Collectively, these studies support an observational association between greater PHD adherence and more favorable cognitive performance or trajectories, but they do not demonstrate that the diet prevents cognitive decline.

### 7.2. Incident Dementia and Alzheimer’s Disease

A separate body of prospective evidence has evaluated clinically diagnosed dementia and AD as incident outcomes. In the Rush Memory and Aging Project, Yang et al. followed 926 older adults for up to 20 years and reported that higher PHD adherence was associated with a lower incidence of dementia and AD and with a later age at dementia diagnosis [26]. Importantly, these findings were derived from an observational cohort and therefore indicate an association rather than evidence that PHD adherence prevents dementia.

In the Malmö Diet and Cancer Study, Samuelsson et al. also reported an inverse association between adherence to an EAT–Lancet-based dietary score and incident dementia over more than 15 years of follow-up [28]. However, the magnitude and consistency of the associations varied according to the dietary scoring approach and APOE ε4 status. These findings highlight both the potential relevance of dietary pattern and the sensitivity of results to exposure definition and genetic susceptibility. Taken together, prospective cohorts provide suggestive evidence that greater adherence to PHD-related dietary patterns is associated with a lower incidence of dementia, but PHD-specific randomized trials with incident dementia or AD as outcomes are currently lacking.

### 7.3. Neuropathological and Neuroimaging Correlates

Beyond clinical and cognitive outcomes, a smaller body of evidence has examined brain pathology and neuroimaging markers. In the Rush Memory and Aging Project, higher PHD adherence was associated with lower postmortem burdens of amyloid plaques and neurofibrillary tangles [26]. These findings are notable because neuropathological assessment provides an objective biological correlate of AD; however, the observational design does not establish that dietary adherence directly altered amyloid or tau pathology.

Neuroimaging studies provide complementary evidence. Samuelsson et al. reported that greater adherence to the EAT–Lancet diet was associated with more favorable brain MRI measures, including indices of white matter integrity and hippocampal volume [56]. Such structural markers may help bridge epidemiological associations with biological processes underlying cognitive aging, but current evidence remains cross-sectional or observational. Longitudinal imaging studies and controlled dietary interventions are needed to determine whether changes in PHD adherence precede measurable changes in brain structure or pathology.

### 7.4. Effect Modification, Biological Aging, and Interpretive Considerations

Several studies suggest that the association between PHD adherence and cognitive outcomes may vary across biological and social contexts. APOE genotype has been examined as a potential effect modifier, with some studies reporting weaker associations among APOE ε4 carriers [28,54]. Socioeconomic status may also influence the observed relationship. Zhao et al. reported that associations between PHD adherence and dementia risk differed across socioeconomic strata, while the ELSA–Brazil study identified income-related heterogeneity in associations with cognitive decline [25,53,55,56,57,58,59,60]. These findings raise the possibility that access to healthy foods, education, healthcare, physical activity opportunities, and other correlated social determinants contribute to the observed diet–cognition relationship.

Biological aging has also been proposed as a potential intermediate pathway. In UK Biobank analyses, higher PHDI adherence was associated with lower risks of several brain disorders, and statistical mediation analyses suggested that measures of biological aging accounted for part of these associations [58]. Such findings are hypothesis-generating, but mediation estimates derived from observational data should not be interpreted as proof of a biological causal pathway. Likewise, associations between PHD adherence and preserved intrinsic capacity or broader neurological outcomes may reflect a combination of dietary exposure, cardiometabolic health, socioeconomic position, and healthy lifestyle behaviors [61,62].

Overall, the consistency of associations across several cohorts is encouraging, but causal interpretation remains limited by residual confounding, heterogeneity in PHD scoring, differences in cognitive outcome definitions, reverse causation, and the absence of PHD-specific randomized trials with cognitive or dementia endpoints.

**Table 5:** Summary of studies examining the association between Planetary Health Diet adherence and cognitive aging outcomes.

Study	Population	Design	Key Finding	Outcome
Yang et al. [26]	Rush MAP, *n* = 926	Prospective, up to 20 y + autopsy	Lower dementia risk, delayed onset, lower AD pathology at autopsy	Dementia, neuropathology
Gomes Gonçalves et al. [25]	ELSA–Brazil, *n* = 11,737	Prospective, ~8 y	Slower memory and global cognitive decline; dose-response; effect modification by income	Memory, global cognition
Samuelsson et al. [28]	Malmö Diet & Cancer	Prospective, 15+ y	Lower incident dementia; associations varied by score and APOE ε4	Incident dementia
Tang et al. [27]	Chinese older adults	Prospective cohort	Slower cognitive decline with higher PHD adherence	Cognitive decline trajectory
Chen et al. [53]	3 US cohorts, *n* = 159,347	Prospective	Lower subjective cognitive decline risk	Subjective cognitive decline
Zhang et al. [54]	Singapore Chinese	Prospective	PHD–cognition link stronger in APOE ε4 non-carriers	Late-life cognitive function
Li et al. [57]	Chinese older adults	Cross-sectional/prospective	Lower cognitive impairment risk; role of animal-source components	Cognitive impairment
Li et al. [58]	UK Biobank	Prospective	Lower brain disorder risk; biological aging partially mediates	Brain disorders, biological aging
van Soest et al. [55]	UK Biobank	Cross-sectional	Better cognitive aging with EAT–Lancet adherence	Cognitive aging markers
Samuelsson et al. [56]	Swedish cohort	Cross-sectional	Better brain MRI markers (white matter, hippocampal volume)	Brain MRI markers
Zhao et al. [60]	Population-based	Prospective	PHD–dementia association moderated by SES	Incident dementia
Shi et al. [61]	Multicohort	Prospective	Plant-forward patterns lower risk of major brain disorders	Multiple brain disorders
Gómez-Ca et al. [62]	Older adult cohort	Cross-sectional	PHD associated with preserved intrinsic capacity	Intrinsic capacity

**Figure 3:**
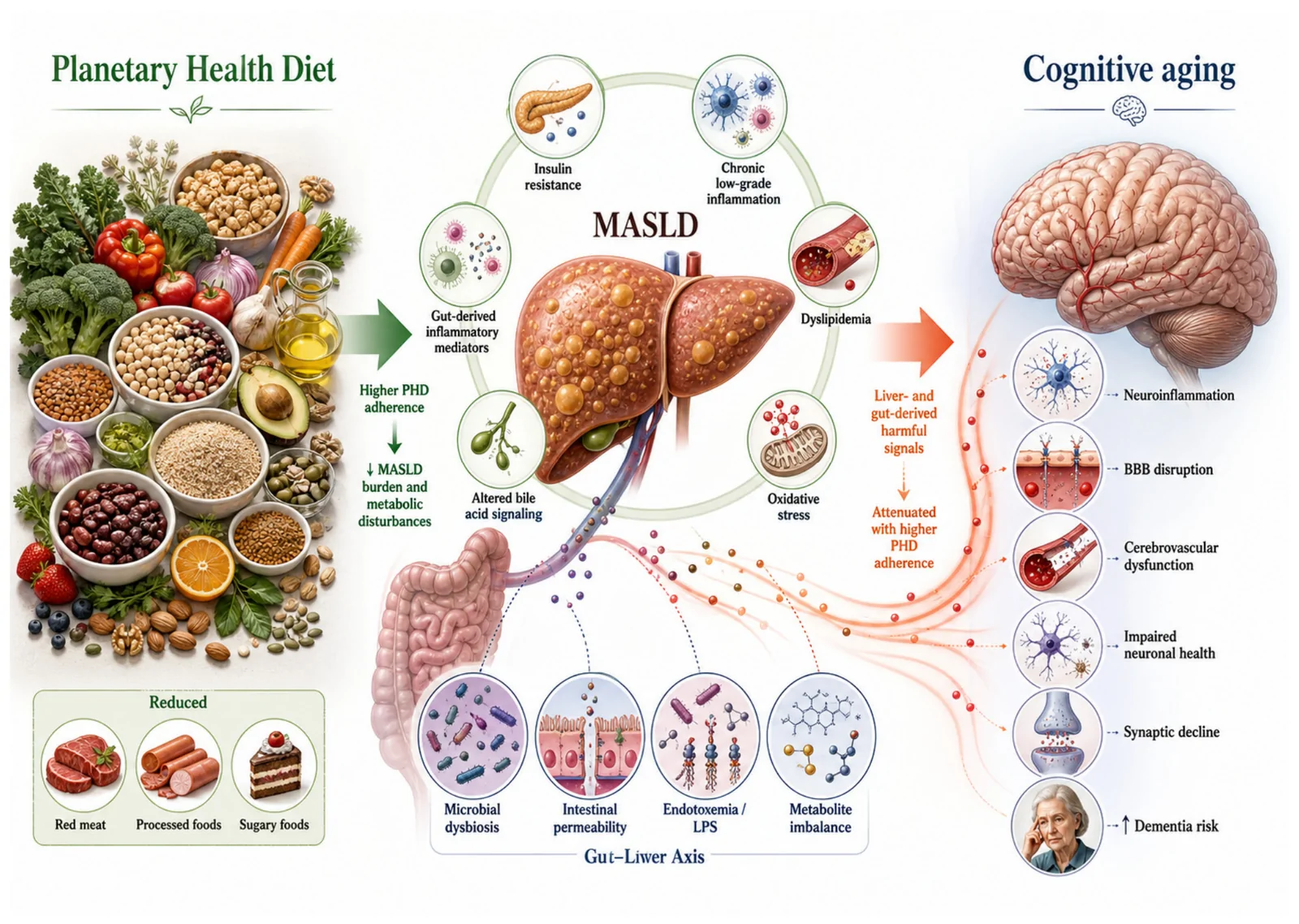
Conceptual model of MASLD as a potential metabolic amplifier within the proposed PHD–gut–liver–brain framework. Solid relationships represent comparatively better-supported associations, whereas proposed mechanistic connections should be interpreted as hypothesis-generating where direct PHD-specific human evidence is unavailable. MASLD, metabolic dysfunction-associated steatotic liver disease; PHD, Planetary Health Diet; BBB, blood–brain barrier; SCFAs, short-chain fatty acids; LPS, lipopolysaccharide.

Figure 3 presents a mechanistic framework illustrating how MASLD may act as a metabolic amplifier within the gut–liver–brain axis, linking the Planetary Health Diet to cognitive aging outcomes through shared inflammatory, metabolic, and microbial pathways.

The distinction between these outcome domains is important. Associations with cognitive performance or longitudinal cognitive decline provide evidence concerning trajectories of cognitive aging; associations with incident dementia or AD address clinically defined disease outcomes; and neuropathological or neuroimaging findings provide biological correlates rather than clinical evidence of prevention. None of these observational evidence streams, individually or collectively, establishes that PHD adherence prevents dementia. Rather, they provide convergent but still non-causal evidence that supports further testing in intervention studies.

## 8. The Gut–Liver–Brain Axis: Mechanistic Pathways and Levels of Evidence

The gut–liver–brain axis represents an integrated network of bidirectional communication among the gastrointestinal tract, liver, and central nervous system through portal circulation, bile acid enterohepatic cycling, neural signaling, immune mediators, and gut-derived metabolites [9,10]. The PHD is biologically relevant to several components of this network because it emphasizes fiber-rich plant foods, unsaturated fats, and limited intake of highly processed foods. However, the mechanistic evidence reviewed in this section derives from different levels of investigation and should not be considered equivalent. Direct human evidence demonstrating that adherence to the complete PHD modifies gut–liver–brain signaling remains limited. Some mechanistic support comes from human studies of Mediterranean or other plant-forward diets, whereas additional evidence is derived from individual dietary components, microbial metabolites, experimental systems, and preclinical models. Accordingly, the pathways discussed below are classified as directly observed, indirectly supported, or biologically plausible according to the source of evidence.

### 8.1. The Gut Microbiome as a Central Hub

Diet is the single most important modifiable determinant of gut microbiome composition and function [63]. The human gut harbors trillions of microorganisms whose collective metabolic output profoundly influences host physiology, including nutrient metabolism, immune development and regulation, intestinal barrier maintenance, and endocrine signaling. Plant-based and plant-forward diets have been consistently shown to increase microbial diversity and promote the abundance of short-chain fatty acid (SCFA)-producing bacteria, including Faecalibacterium prausnitzii, Roseburia species, and Eubacterium rectale [64,65]. The PHD’s high content of dietary fiber from whole grains, legumes, vegetables, and fruits provides abundant substrates for microbial fermentation, while its limited processed food content reduces exposure to emulsifiers, artificial sweeteners, and other additives that may disrupt microbial communities [66]. Mediterranean-type diets rich in plant foods have been shown to improve intestinal inflammation markers, increase microbial diversity, and favor beneficial metabolite production [67,68]. Since the PHD shares substantial overlap with Mediterranean dietary principles, similar microbiome-mediated benefits are biologically plausible. Indeed, the interplay between diet and the gut microbiome is now recognized as a key determinant of metabolic and intestinal health, with different dietary patterns profoundly shaping microbial community composition and function [63]. In the context of MASLD, gut dysbiosis—characterized by reduced microbial diversity, decreased SCFA-producing bacteria, increased Proteobacteria, and elevated fecal *Escherichia coli*—has been linked to disease onset and progression [39,69]. Human dietary studies indicate that plant-based and plant-forward dietary patterns can influence gut microbial composition and metabolite production, including enrichment of several SCFA-producing taxa [63,64,65]. Mediterranean-type dietary interventions have also been associated with changes in microbial diversity, intestinal inflammatory markers, and microbial metabolites [67,68]. These findings provide indirect mechanistic support for the PHD because the PHD shares several dietary characteristics with these patterns, particularly its high intake of whole grains, legumes, vegetables, fruits, and nuts.

However, direct evidence demonstrating that adherence to the complete PHD restores a specific dysbiotic microbiome signature in individuals with MASLD or cognitive impairment is currently limited. Therefore, microbiome modulation should be regarded as a biologically plausible pathway supported primarily by related dietary pattern studies rather than as a PHD-specific mechanism established in humans.

### 8.2. Short-Chain Fatty Acids: From Dietary Fiber to Brain Protection

Short-chain fatty acids—principally acetate, propionate, and butyrate—are the primary metabolites of microbial dietary fiber fermentation and represent a critical interface between diet, gut health, and systemic physiology [70,71]. Butyrate serves as the principal energy source for colonocytes and plays a pivotal role in maintaining intestinal barrier integrity by promoting tight junction protein expression, stimulating mucus production, and supporting epithelial cell proliferation. In addition, butyrate functions as a potent histone deacetylase (HDAC) inhibitor, modulating gene expression in both intestinal epithelial cells and immune cells to promote anti-inflammatory programs [70,72]. Propionate is transported to the liver via the portal circulation, where it influences hepatic gluconeogenesis regulation, lipogenesis suppression, and appetite regulation through activation of G-protein-coupled receptors GPR41 and GPR43 [73]. Acetate, the most abundant SCFA, enters systemic circulation and influences peripheral tissues including adipose tissue, muscle, and brain.

Human intervention evidence directly linking the complete PHD to changes in circulating or fecal SCFAs is currently sparse. Evidence from related dietary patterns provides partial support: Mediterranean dietary interventions, including the LIBRE trial, have reported changes in SCFA-related metabolism and intestinal barrier measures [74]. These findings suggest that fiber-rich dietary patterns can modify microbial fermentation products in humans, but they should not be interpreted as direct evidence of a PHD-specific SCFA response.

Evidence connecting SCFAs with brain immune responses is even less direct. Experimental and disease-model studies have implicated SCFAs in microglial activity, cytokine signaling, and neuroinflammatory responses [75]. Such findings provide biological plausibility for a gut-derived route linking dietary fiber to brain function, but they do not establish that PHD-induced increases in SCFAs alter microglial polarization or cognitive outcomes in humans. Similarly, SCFAs have been implicated in hepatic energy and lipid metabolism, providing a plausible link to MASLD. Thus, SCFAs are best regarded as candidate mediators connecting dietary fiber, microbial metabolism, liver function, and brain signaling rather than as a clinically demonstrated mechanism of the PHD.

### 8.3. Bile Acid Signaling and the Putative FXR/TGR5–Brain Pathway

Bile acids occupy a unique position at the intersection of lipid metabolism, gut microbial ecology, and signaling physiology. Primary bile acids (cholic acid and chenodeoxycholic acid) are synthesized in the liver from cholesterol, conjugated with glycine or taurine, secreted into the intestine as part of bile, and subsequently modified by gut microbiota through deconjugation and 7α-dehydroxylation to generate secondary bile acids (deoxycholic acid and lithocholic acid) [41]. This microbial biotransformation is critical because secondary bile acids have distinct signaling properties compared with their primary precursors.

Bile acids function not merely as lipid-digesting detergents but as potent signaling molecules acting through two principal receptors: the nuclear farnesoid X receptor (FXR) and the membrane-bound Takeda G-protein-coupled receptor 5 (TGR5) [76,77]. FXR signaling regulates hepatic lipid metabolism, bile acid homeostasis, glucose metabolism, and inflammatory responses, with FXR activation generally exerting anti-inflammatory and metabolic-protective effects in the liver. TGR5 signaling modulates energy expenditure, glucose homeostasis, and immune cell function, with TGR5 activation in macrophages and monocytes promoting an anti-inflammatory phenotype [77].

Alterations in bile acid composition and FXR/TGR5 signaling have been implicated in metabolic liver disease, and experimental studies support roles for these receptors in lipid metabolism, glucose homeostasis, and inflammatory regulation [76,77,78]. Evidence for a corresponding bile acid–brain signaling pathway is emerging but remains substantially less developed, particularly in humans. Proposed mechanisms include systemic bile acid signaling, receptor-mediated immune modulation, and neural communication, but these pathways have not been directly demonstrated as consequences of PHD adherence.

Accordingly, the hypothesis that the PHD modifies cognitive aging through microbiome-dependent changes in bile acid composition and subsequent FXR/TGR5 signaling should currently be regarded as mechanistically plausible rather than clinically established. PHD-specific intervention studies incorporating bile acid profiling and neurological outcomes will be required to determine whether this pathway contributes meaningfully to the observed epidemiological associations.

### 8.4. Intestinal Barrier Integrity and Metabolic Endotoxemia

The intestinal barrier is a dynamic, highly regulated system comprising a single layer of columnar epithelial cells sealed by tight junction proteins (claudins, occludin, zonula occludens), overlaid by a mucus layer containing mucins and antimicrobial peptides, and further protected by the luminal microbiota through colonization resistance [79]. Under conditions of gut dysbiosis, this barrier can be compromised, leading to increased intestinal permeability—colloquially termed “leaky gut”—and translocation of bacterial products including lipopolysaccharide (LPS), peptidoglycan fragments, and bacterial extracellular vesicles into the portal and systemic circulation [39].

Low-grade metabolic endotoxemia, characterized by chronically elevated circulating LPS levels, activates Toll-like receptor 4 (TLR4) on hepatocytes, Kupffer cells, and circulating monocytes, triggering NF-κB-dependent production of pro-inflammatory cytokines including tumor necrosis factor-α, interleukin-6, and interleukin-1β [80]. In the liver, this endotoxin-driven inflammatory cascade promotes hepatic inflammation, insulin resistance, and progression from simple steatosis to MASH and fibrosis [9,40]. The portal circulation provides a direct anatomical conduit for gut-derived endotoxins and metabolites to reach the liver, making the gut–liver axis particularly vulnerable to intestinal barrier disruption.

Emerging evidence suggests that gut-derived metabolites and bacterial extracellular vesicles can also reach the brain through systemic circulation, potentially influencing neuroinflammatory processes, blood–brain barrier integrity, and neuronal function [9,10]. In MASLD patients, chronic low-grade endotoxemia may contribute to both hepatic inflammation and subclinical neuroinflammation, providing a mechanistic bridge between liver disease and cognitive impairment. Experimental and clinical research outside the PHD-specific literature supports a relationship among dietary fiber, microbial metabolites, intestinal barrier function, and circulating endotoxin exposure [39,40,79,80]. In MASLD, increased intestinal permeability and endotoxemia have been associated with hepatic inflammatory processes, while broader gut–brain research suggests that microbial products and metabolites may also influence neuroimmune signaling. However, direct human evidence showing that PHD adherence reduces intestinal permeability or LPS translocation and consequently improves both liver and cognitive outcomes is not currently available. Therefore, preservation of intestinal barrier integrity represents an indirect mechanistic hypothesis supported by the known effects of dietary fiber and related dietary patterns rather than a demonstrated PHD-specific effect.

### 8.5. Liver–Brain Neuroinflammatory Crosstalk

The liver–brain axis represents a bidirectional communication network involving neural (vagal and spinal afferent), metabolic (ammonia, lactate, aromatic amino acids), inflammatory (cytokines, chemokines), and hormonal (insulin, adipokines) pathways [8]. In MASLD, chronic hepatic inflammation generates systemic inflammatory mediators that can disrupt blood–brain barrier integrity, promote microglial activation, and impair neuronal function, even in the absence of overt hepatic encephalopathy [7]. A systematic review and meta-analysis by Wang et al. estimated that NAFLD was associated with a 1.3-fold increased risk of dementia or cognitive impairment, establishing an epidemiological link between fatty liver disease and brain health [6].

The mechanisms linking MASLD to cognitive impairment are likely multifactorial and may include subclinical hepatic encephalopathy, chronic systemic inflammation, vascular dysfunction and cerebrovascular disease, insulin resistance in the brain, and altered bile acid and ammonia metabolism [7,8]. Metabolic inflammation—a concept encompassing the chronic, low-grade inflammatory state associated with obesity, T2D, and metabolic syndrome—provides a unifying framework linking these conditions to neurodegeneration [81]. The microbiota–gut–brain axis further integrates these pathways through vagal nerve signaling, immune mediators, and microbial metabolites including SCFAs, tryptophan metabolites, and bile acids that influence brain inflammation and function [10].

The epidemiological association between MASLD and cognitive impairment provides a clinical context for considering liver–brain crosstalk, but it does not establish that hepatic inflammation is the direct cause of cognitive decline. Shared cardiometabolic and vascular factors, including obesity, diabetes, hypertension, and systemic atherosclerotic disease, may contribute substantially to both conditions, as discussed above.

Within this context, the PHD could theoretically influence several upstream determinants shared by liver and brain health, including dietary quality, insulin sensitivity, inflammatory tone, and gut microbial metabolism. Nevertheless, no study identified in this review directly demonstrated that a PHD intervention modifies a defined liver–brain inflammatory pathway and subsequently improves cognitive outcomes. The proposed PHD–gut–liver–brain model should therefore be understood as an integrative hypothesis assembled from observational associations, human evidence from related dietary patterns, and mechanistic findings from component-level and experimental studies.

Overall, current mechanistic evidence supports the biological plausibility of interactions among diet, the gut microbiome, liver metabolism, and brain function, but the degree of direct PHD-specific evidence varies substantially across pathways. Evidence is comparatively stronger for general diet–microbiome and gut–liver relationships, whereas SCFA-mediated neuroimmune effects, FXR/TGR5-related brain signaling, and integrated liver–brain inflammatory pathways rely more heavily on component-level, experimental, or non-PHD dietary evidence. These distinctions are essential when interpreting the observational associations between PHD adherence, MASLD, and cognitive outcomes and identify clear priorities for future mechanistic intervention studies.

## 9. Clinical Implications and Practical Considerations

The clinical interpretation of the PHD requires a clear distinction between its original role as a population-level framework for healthy and environmentally sustainable eating and the concept of a clinically prescribed therapeutic diet. The PHD was not originally developed as a disease-specific intervention for MASLD, cognitive impairment, or dementia, and current evidence does not justify treating it as a standardized therapeutic prescription for these conditions. Rather, its principal clinical relevance lies in the extent to which its core features—greater consumption of minimally processed plant foods, whole grains, legumes, nuts, fruits, and vegetables, together with lower intake of red and processed meat and added sugars—overlap with established principles of cardiometabolic health.

Accordingly, PHD principles may inform dietary counseling at the population and individual levels, but clinical implementation should remain individualized according to disease status, nutritional requirements, age, frailty, comorbidities, cultural context, food availability, and treatment goals. For hepatology and gastroenterology practice, the growing PHD-MASLD evidence supports incorporating dietary quality assessment, including PHDI or similar indices, into routine MASLD management alongside established recommendations for weight loss, physical activity, and cardiometabolic risk reduction [2]. For patients with MASLD, dietary counseling may reasonably emphasize overall dietary quality alongside established management priorities such as weight control, physical activity, and cardiometabolic risk reduction. Similarly, in older adults with cognitive concerns, attention to metabolic health and nutritional status may be clinically relevant given the overlap in cardiometabolic risk factors between MASLD and cognitive aging. However, the current evidence does not establish that PHD adherence should replace guideline-based MASLD management or that PHD-specific dietary counseling constitutes a validated dementia-prevention strategy.

The PHD shares substantial overlap with the Mediterranean diet, which has the most robust evidence base for both MASLD management and cognitive health. Because the PHD shares substantial features with the Mediterranean diet, clinicians may draw on overlapping food-based principles—such as greater intake of legumes, whole grains, nuts, vegetables, fruits, fish, and unsaturated fats and lower intake of red and processed meat—when translating the PHD concept into practical dietary advice. However, evidence supporting Mediterranean dietary interventions should not be assumed to constitute direct evidence for the PHD itself. Specific dietary counseling should focus on practical food substitutions: replacing processed meats with legumes, fish, poultry, or plant-based protein sources; replacing refined grains with whole grains; replacing sugar-sweetened beverages with water or unsweetened alternatives; increasing vegetable and legume portions at meals; and using unsaturated plant oils in place of saturated fats.

Nutritional adequacy requires particular attention in older adults, especially those with frailty, reduced appetite, or sarcopenia. A plant-forward diet should not be implemented in a manner that compromises total energy or protein intake. Adequate protein quantity and quality are important for preservation of muscle mass and physical function, and protein should be distributed across meals using a combination of plant and, where appropriate, animal-source foods. Legumes, soy foods, nuts, seeds, and whole grains can provide complementary plant protein, while dairy products, eggs, fish, or poultry may contribute additional high-quality protein depending on individual dietary preferences and cultural context. In older adults with established sarcopenia or high nutritional risk, individualized assessment by a qualified clinician or dietitian is preferable to rigid application of population-level PHD targets [66], as shwon in Table 6.

Potential micronutrient insufficiency represents another important consideration. Modeling studies have suggested that strict implementation of the EAT–Lancet reference diet may result in inadequate intakes of vitamin B12, calcium, iron, and zinc in some populations, particularly where access to fortified foods or supplements is limited [13]. These concerns are especially relevant in older adults because deficiencies may coexist with anemia, impaired bone health, reduced physical function, or other age-related vulnerabilities. Therefore, supplementation should not be recommended uniformly but should be considered according to dietary intake, biochemical assessment, clinical status, and local practice. Fortified foods or targeted supplementation may be appropriate where a specific deficiency or high risk of deficiency is identified.

Weight management remains a cornerstone of MASLD management, with even modest (5–10%) weight loss shown to improve hepatic steatosis, inflammation, and in some cases fibrosis. Physical activity, both aerobic and resistance-based, provides synergistic benefits by enhancing gut microbial diversity, improving insulin sensitivity, promoting weight maintenance, and independently supporting cognitive function through neurotrophin upregulation and cerebrovascular health. Dietary strategies should be integrated with, rather than viewed as substitutes for, guideline-directed management of obesity, diabetes, dyslipidemia, and other cardiometabolic risk factors [76].

In summary, the PHD is best viewed clinically as a flexible dietary framework whose principles may complement established nutritional and cardiometabolic care, rather than as a standardized therapeutic diet for MASLD or cognitive decline. Translation into clinical practice should prioritize nutritional adequacy, preservation of muscle mass, cultural adaptability, affordability, and individual disease context.

## 10. Challenges and Limitations

Despite the growing body of evidence linking PHD adherence to MASLD and cognitive outcomes, several critical challenges and limitations must be acknowledged. First, and most fundamentally, the existing evidence is predominantly observational. Prospective cohort studies, cross-sectional analyses, and ecological comparisons cannot establish causality, and residual confounding by unmeasured or imprecisely measured variables remains a persistent concern. Several limitations should be considered when interpreting the current literature. Most importantly, the evidence linking PHD adherence with MASLD and cognitive outcomes remains predominantly observational. Prospective cohort studies can establish temporality more effectively than cross-sectional analyses, but they cannot fully eliminate residual confounding. Higher PHD adherence frequently clusters with lower adiposity, greater physical activity, lower smoking prevalence, higher educational attainment, greater health literacy, higher socioeconomic position, and better access to preventive healthcare. Many of these characteristics are independently associated with both liver and cognitive health. Consequently, part of the observed PHD–outcome association may reflect a broader healthy-user and socioeconomic pattern rather than the effect of diet alone [60].

Socioeconomic status may additionally operate as an effect modifier rather than solely as a confounder, because affordability, food availability, educational resources, healthcare access, and the capacity to sustain a high-quality diet differ across social strata. The stronger associations reported in some higher-SES groups should therefore not automatically be interpreted as evidence of greater biological responsiveness to the PHD.

A second major limitation is the scarcity of PHD-specific interventional evidence. Although randomized trials of related healthy dietary patterns provide useful contextual evidence, findings from Mediterranean, DASH, or MIND interventions cannot be assumed to demonstrate the effects of the PHD itself. For example, the MIND randomized trial showed improvement in cognition in both the intervention and control groups, with no statistically significant between-group difference [31]. At present, the PHD-specific evidence summarized in this review is therefore insufficient to establish that increasing PHD adherence causes improvement in MASLD or prevents cognitive decline or dementia. Long-term dietary trials are inherently challenging because of difficulties in participant blinding, adherence, dietary contamination between groups, and selection of appropriate comparator diets. Third, the PHD was developed primarily as a framework integrating human health with environmental sustainability rather than as a disease-specific therapeutic diet for MASLD or neurodegenerative disorders. Consequently, its food-group targets should not be assumed to represent optimal clinical prescriptions for all patients. This distinction is particularly important in older adults with frailty, sarcopenia, multimorbidity, altered energy requirements, or micronutrient deficiencies, for whom rigid adherence to population-level targets could conflict with individualized nutritional needs. The environmental and health objectives of the PHD are complementary in principle but may not always translate into identical dietary priorities at the individual clinical level. Fourth, the feasibility and meaning of PHD adherence vary substantially across cultural and socioeconomic settings. The availability, affordability, preparation, and cultural acceptability of legumes, nuts, whole grains, dairy products, fish, and plant-based alternatives differ across geographic regions and income groups. A dietary pattern that is readily achievable in one population may require substantial financial, culinary, or structural resources in another. Consequently, observed associations between PHD adherence and health outcomes may partly capture social advantage and food-environment differences. Future PHD adaptations should therefore preserve the underlying dietary principles while allowing culturally appropriate, locally available, and economically feasible food substitutions.

Fifth, substantial methodological heterogeneity limits direct comparison across studies. The term “PHD adherence” encompasses several operational scoring systems that differ in food-group definitions, cut-points, weighting schemes, and the use of absolute versus population-relative intake criteria. Thus, apparently similar exposure categories may not represent equivalent dietary patterns across cohorts. Heterogeneity is further introduced by differences in MASLD ascertainment, cognitive testing, dementia definitions, neuroimaging outcomes, dietary assessment instruments, and duration of follow-up. These differences limit quantitative synthesis and may contribute to variability in reported effect estimates.

Reverse causation represents an additional concern, particularly in cross-sectional studies and cohorts with short intervals between dietary assessment and outcome ascertainment. Early cognitive impairment may influence food purchasing, meal preparation, appetite, and dietary reporting, creating an apparent association between poorer diet quality and cognitive status. Similarly, individuals diagnosed with MASLD, obesity, or diabetes may improve their diets in response to medical advice, potentially weakening, reversing, or otherwise distorting the observed relationship between dietary scores and metabolic outcomes. Longer prospective follow-up, repeated dietary measurements, and exclusion of early outcome events may help reduce—but cannot completely remove—this source of bias.

Finally, composite adherence scores make it difficult to identify which individual PHD components, combinations of foods, or substitutions account for the observed associations. A higher score may arise through different dietary pathways—for example, greater intake of legumes and whole grains, lower red-meat intake, reduced added sugars, or combinations of these changes—yet these patterns may not be biologically equivalent. Mechanistic interpretation is further limited because much of the evidence concerning SCFAs, intestinal permeability, bile acid signaling, FXR/TGR5 pathways, and neuroimmune regulation originates from individual nutrients, related dietary patterns, or experimental models rather than from controlled PHD interventions. Consequently, the integrated PHD–gut–liver–brain framework proposed in this review should be viewed as a biologically plausible synthesis that remains to be validated through mechanistically informed human intervention studies.

Taken together, these limitations do not negate the consistency of the emerging observational literature, but they constrain causal interpretation and underscore the need to distinguish epidemiological associations from experimentally demonstrated dietary effects.

## 11. Future Directions

Several research priorities emerge from the current evidence. The highest priority is the development of well-designed PHD-specific intervention studies that can move the field beyond observational associations. Randomized controlled trials should evaluate whether culturally adapted PHD interventions produce measurable changes in liver and cognitive outcomes relative to appropriate comparator diets. Trials incorporating complementary endpoints—such as MRI–proton density fat fraction (MRI-PDFF), validated cognitive composite scores, cardiometabolic biomarkers, and repeated dietary assessments—would be particularly informative. Adequate intervention duration, adherence assessment, intention-to-treat analysis, and longer-term follow-up will be important for distinguishing transient dietary responses from sustained clinical effects.

Causal inference approaches may complement intervention research but should be interpreted cautiously. Mendelian randomization and related methods could strengthen causal inference regarding cardiometabolic pathways relevant to PHD-like dietary exposures, although valid genetic instruments for complex dietary patterns remain challenging. Triangulation across prospective cohorts, intervention studies, genetic analyses, and natural experiments may therefore provide more informative evidence than reliance on any single design [22].

Mechanistically informed intervention studies represent a second major priority. Integration of gut microbiome profiling with metabolomics, bile acid and SCFA measurements, lipidomics, inflammatory biomarkers, and other omics platforms could help determine which components of the proposed gut–liver–brain framework actually change in response to PHD exposure. Repeated sampling before, during, and after intervention would be particularly valuable for separating baseline cross-sectional differences from diet-induced biological responses. Neuroimaging substudies incorporating hippocampal volume, cortical structure, white matter integrity, or other validated brain markers could further clarify whether dietary changes are accompanied by measurable changes in brain-related phenotypes.

Future studies should also examine heterogeneity in dietary associations and responses. Genetic susceptibility, including APOE and MASLD-related variants, baseline metabolic phenotype, microbiome composition, age, sex, frailty, and socioeconomic context may modify associations with PHD adherence. Precision-nutrition approaches integrating these factors may eventually support more individualized dietary strategies, but this objective should follow rather than precede demonstration of reproducible PHD-specific effects in controlled human studies.

Implementation research is equally important because the feasibility of the PHD depends on food availability, affordability, cultural preferences, and local food systems. Pragmatic studies should evaluate culturally adapted versions of the PHD across diverse populations while preserving its underlying principles rather than imposing identical food-group targets across settings. Future work should also assess adherence, acceptability, nutritional adequacy, environmental outcomes, and economic implications. Digital dietary assessment tools may facilitate repeated measurement and improve adherence monitoring, although their added value should be formally evaluated.

Fourth, multi-omics studies integrating shotgun metagenomics, metatranscriptomics, metabolomics (including SCFA and bile acid profiling), lipidomics, proteomics, and inflammatory biomarker panels should be incorporated into dietary intervention studies to identify PHD-responsive biomarkers and elucidate mechanistic pathways. Repeated sampling would enable distinction between baseline associations and diet-induced changes. Fifth, neuroimaging substudies examining PHD effects on brain structure (hippocampal volume, cortical thickness, white matter hyperintensity burden) and function (functional connectivity, cerebral blood flow) would strengthen the evidence linking dietary patterns to brain health endpoints.

Sixth, digital health tools for dietary monitoring, including smartphone applications, image-based food recognition, and wearable biosensors, may improve dietary assessment precision and enable real-time feedback to support PHD adherence. Seventh, implementation research is needed to develop culturally adapted PHD versions for diverse global populations, assess their affordability and food security implications, and evaluate their health effects using pragmatic trial designs. Economic evaluation of PHD-based interventions for dementia and MASLD prevention—including cost-effectiveness modeling and budget impact analyses—would provide essential evidence for healthcare policymakers. Finally, gut microbiome-targeted interventions including prebiotics, probiotics, fermented foods, and potentially fecal microbiota transplantation should be explored as adjuncts to PHD-based dietary strategies for enhancing gut–liver–brain axis function.

**Figure 4:**
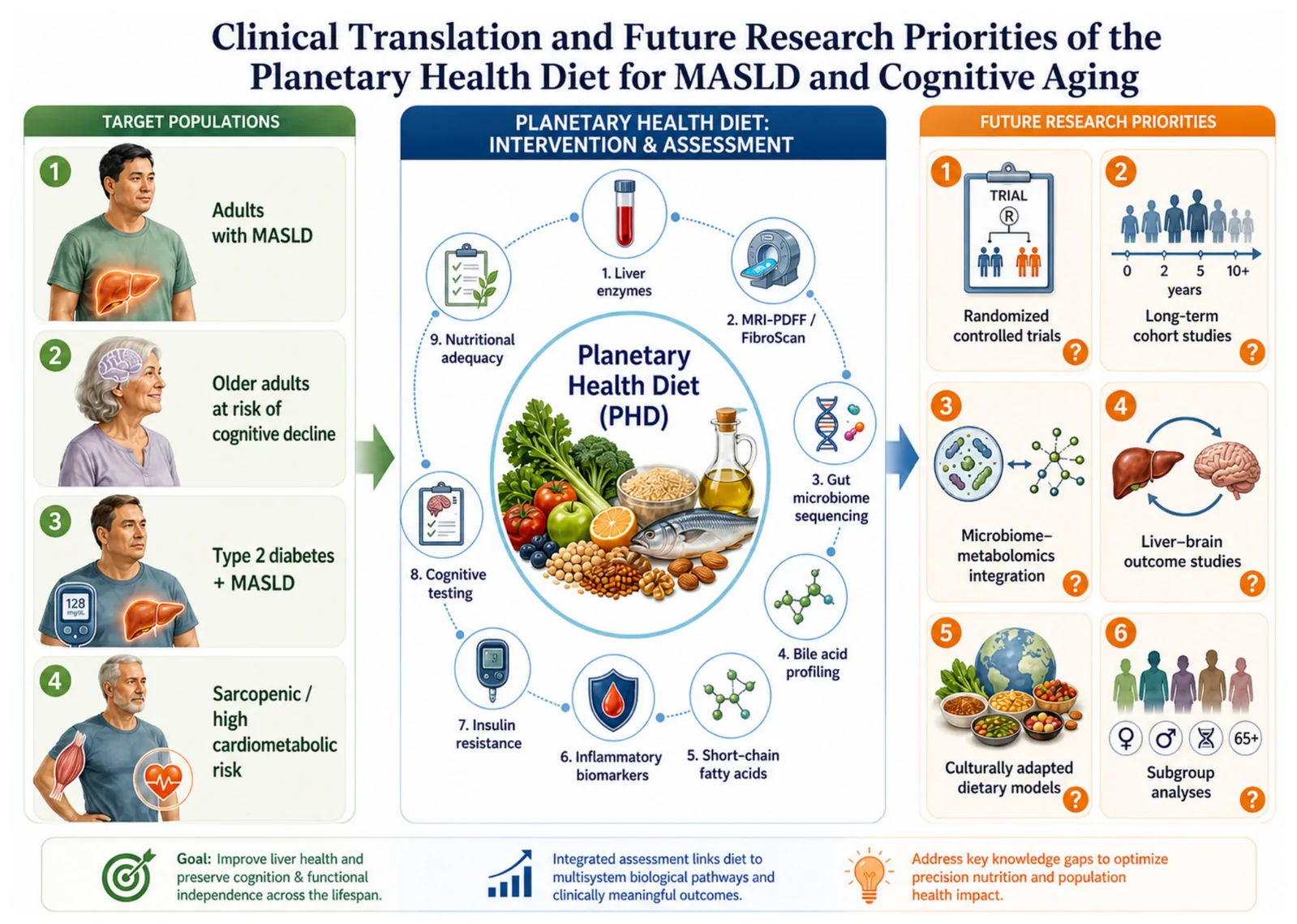
Clinical translation and future research priorities for the Planetary Health Diet in MASLD and cognitive aging. PHD, Planetary Health Diet; MASLD, metabolic dysfunction-associated steatotic liver disease; RCT, randomized controlled trial; MRI-PDFF, magnetic resonance imaging–proton density fat fraction.

Figure 4 summarizes the key clinical translation pathways and future research priorities for PHD-based interventions targeting MASLD and cognitive aging.

## 12. Conclusions

The Planetary Health Diet provides a useful framework for considering the intersection of dietary quality, environmental sustainability, metabolic health, and healthy aging. Current observational evidence consistently suggests that greater adherence to PHD-related dietary patterns is associated with more favorable MASLD outcomes and with several dimensions of cognitive aging, including cognitive performance, longitudinal cognitive trajectories, incident dementia, and selected neuropathological or neuroimaging markers. However, these outcome domains represent distinct levels of evidence and should not be interpreted collectively as proof that the PHD prevents MASLD, cognitive decline, or dementia.

The gut–liver–brain axis provides a biologically plausible framework for integrating these epidemiological observations. Dietary fiber, microbial metabolites, intestinal barrier function, bile acid signaling, systemic inflammation, and cardiometabolic pathways may contribute to shared liver–brain biology. Nevertheless, the evidence supporting these pathways varies substantially in directness. Some relationships are supported by human dietary studies, whereas others—including SCFA-mediated neuroimmune effects and FXR/TGR5-related brain signaling—remain dependent largely on evidence from individual dietary components, related dietary patterns, or experimental models. Direct demonstration that PHD-induced changes in these pathways mediate improvements in both liver and cognitive outcomes is still lacking.

Accordingly, the current value of the PHD in this context lies less in its status as an established therapeutic diet and more in its capacity to provide a testable, integrative framework linking sustainable dietary patterns with metabolic, microbial, hepatic, and cognitive health. Future PHD-specific randomized trials incorporating liver imaging, cognitive assessment, microbiome and metabolomic profiling, and rigorous assessment of nutritional adequacy are needed to determine whether the observed associations translate into clinically meaningful effects. Until such evidence is available, the PHD should be regarded as a promising population-level dietary framework whose potential relevance to MASLD and cognitive aging warrants further mechanistic and interventional investigation.

## Figures and Tables

**Table 1:** Comparison of the Planetary Health Diet with other evidence-based dietary patterns.

Feature	PHD	Mediterranean	DASH	MIND
Origin	EAT–Lancet Commission, 2019 [11]	Observational epidemiology, Seven Countries Study	NIH-funded RCTs for hypertension	Observational cohort analysis [30]
Primary aim	Human health within planetary boundaries	Cardiometabolic health	Blood pressure reduction	Neurodegeneration prevention
Scoring	PHDI, 0–150 points, 16 components [11]	Various indices (MDS, MEDAS, etc.)	DASH score	MIND score, 0–15 points
Grains emphasis	Whole grains: 232 g/d	Whole grains preferred	Whole grains emphasized	Whole grains: ≥3 servings/d
Legumes	75 g/d	Regular consumption	Included	Not specific target
Fish	28 g/d	2–3 servings/week	≤6 oz/week cooked	≥1 serving/week
Added sugars	≤31 g/d	Moderate	Limited	≤5 servings/week
Environmental integration	Yes (explicit)	No	No	No
Cognitive outcomes	Emerging evidence	Meta-analytic evidence	Limited direct evidence	RCT evidence (MIND trial) [31]
Liver outcomes	Growing observational evidence	Well-established	Limited	Very limited

**Table 2:** Shared and putative biological pathways linking MASLD with cognitive aging.

Mechanism	Role in MASLD	Relevance to Cognitive Aging
Lipotoxicity	Excess free fatty acids and toxic lipid species cause hepatocyte injury, activate NF-κB and JNK signaling, promote inflammation and apoptosis [36]	Lipid accumulation in brain cells may impair synaptic function, promote neuroinflammation
Insulin resistance	Enhanced hepatic de novo lipogenesis, impaired β-oxidation, net hepatic lipid accumulation [36]	Brain insulin resistance impairs glucose metabolism, synaptic plasticity, and amyloid clearance
Mitochondrial dysfunction	Impaired oxidative phosphorylation, increased reactive oxygen species, hepatocyte injury [37]	Mitochondrial dysfunction in neurons reduces ATP production, accelerates neurodegeneration
Chronic low-grade inflammation	Elevated TNF-α, IL-6, IL-1β; NF-κB activation; hepatic and systemic inflammatory milieu [38]	Systemic inflammation disrupts blood–brain barrier, promotes microglial activation
Gut dysbiosis & endotoxemia	Altered microbiota, increased intestinal permeability, LPS translocation, portal endotoxemia [39,40]	Microbial metabolites and endotoxins reach brain via circulation, modulate neuroinflammation
Altered bile acid signaling	Dysregulated FXR/TGR5 pathways, altered primary-to-secondary bile acid ratios [41]	Bile acids modulate brain inflammation and metabolism through FXR/TGR5 signaling
Oxidative stress	Lipid peroxidation, mitochondrial ROS production, antioxidant depletion [37]	Oxidative damage to neurons, DNA, and myelin accelerates brain aging
Vascular dysfunction	Endothelial dysfunction, atherosclerosis, increased cardiovascular risk [35]	Cerebrovascular disease contributes to vascular cognitive impairment

**Table 6:** Practical considerations for adapting Planetary Health Diet principles in older adults and individuals with metabolic risk.

Goal	Recommendation	Key Consideration
Increase dietary fiber	Target 25–30 g/d from diverse sources: whole grains, legumes, vegetables, fruits, nuts [66]	Increase gradually to avoid gastrointestinal symptoms; adequate fluid intake
Promote microbiome diversity	Consume ≥ 30 different plant foods/week; include fermented foods (yogurt, kimchi, sauerkraut)	Diversity of plant substrates promotes microbial ecosystem resilience
Optimize fat quality	Replace saturated fats with unsaturated plant oils; include omega-3 from fish (2–3 servings/week) and walnuts [51]	Omega-3 fatty acids (DHA/EPA) have anti-inflammatory and neuroprotective roles
Limit red and processed meat	Target ≤ 14 g/d red meat per PHD guidelines; minimize processed meat. Replace with legumes, fish, poultry, or soy-based proteins	Replace with legumes, fish, poultry, or soy-based proteins
Reduce added sugars and ultra-processed foods	Limit sugar-sweetened beverages, confectionery, and ultra-processed snacks	Reduces hepatic de novo lipogenesis and inflammatory dietary load
Ensure micronutrient adequacy	Monitor vitamin B12, calcium, iron, zinc, vitamin D; supplement if indicated [13]	Particularly important for older adults and those limiting animal-source foods
Maintain adequate protein	≥1.0–1.2 g/kg/d for older adults; higher for sarcopenic individuals	Combine plant and animal protein sources; resistance exercise synergy
Manage body weight	Target 5–10% weight loss when indicated; even modest loss improves MASLD	Caloric adequacy must be maintained, especially in frail older adults
Consider gut–liver–brain axis targets	Emphasize prebiotic fibers, polyphenol-rich foods, and fermented products	These may simultaneously support liver, gut, and brain health

## Data Availability

No new data were created or analyzed in this study. Data sharing is not applicable to this article.

## Abbreviations

The following abbreviations are used in this manuscript:

AD	Alzheimer’s disease
BBB	Blood–brain barrier
DASH	Dietary Approaches to Stop Hypertension
DHA	Docosahexaenoic acid
EPA	Eicosapentaenoic acid
FFQ	Food-frequency questionnaire
FXR	Farnesoid X receptor
GBD	Global Burden of Disease
HDAC	Histone deacetylase
HDCA	Hyodeoxycholic acid
HR	Hazard ratio
LPS	Lipopolysaccharide
MASLD	Metabolic dysfunction-associated steatotic liver disease
MASH	Metabolic dysfunction-associated steatohepatitis
MCI	Mild cognitive impairment
MIND	Mediterranean–DASH Intervention for Neurodegenerative Delay
MRI-PDFF	Magnetic resonance imaging proton density fat fraction
NAFLD	Non-alcoholic fatty liver disease
NF-κB	Nuclear factor kappa B
NHANES	National Health and Nutrition Examination Survey
PHD	Planetary Health Diet
PHDI	Planetary Health Diet Index
RCT	Randomized controlled trial
SCFA	Short-chain fatty acid
SES	Socioeconomic status
T2D	Type 2 diabetes
TGR5	Takeda G-protein-coupled receptor 5
TLR4	Toll-like receptor 4
